# Disaster Emotion: When Media Messages Emphasize Self-Interested Responses

**DOI:** 10.3390/bs16040621

**Published:** 2026-04-21

**Authors:** Soyoung Kim, Christopher Stream, Suyeon Lee

**Affiliations:** 1 School of Liberal Arts, Seoul National University of Science and Technology, 172 Gongneung-dong, Nowon-gu, Seoul 01811, Republic of Korea; 2 Greenspun College of Urban Affairs, University of Nevada, Las Vegas, NV 89154, USA; 3 College of Energy and Biotechnology, Seoul National University of Science and Technology, 172 Gongneung-dong, Nowon-gu, Seoul 01811, Republic of Korea

**Keywords:** disaster communication, media discourse, selfishness, self-interest, sentiment analysis, topic modeling

## Abstract

Media coverage of disasters frequently frames self-interested behavior in contrast to collective responsibility and coordinated response. This study aims to explore how such behavior is emotionally constructed in disaster-related media, using a carefully selected corpus of 12 text-centered news articles focusing on selfish behavior. The analysis combines transformer-based sentence-level emotion classification using the tweetnlp RoBERTa model, which predicts 11 emotion categories, with Latent Dirichlet Allocation topic modeling across single-sentence and three-sentence windows in a small purposively selected corpus. Emotion–topic relationships are quantified by weighting emotion probabilities by topic distributions and visualized using bar charts, network graphs, and heatmaps. The findings suggest that fear and disgust dominate portrayals of self-interested behavior, while anticipation appears in projections of harm and anger is linked to inequality and institutional accountability. Two discursive configurations emerge: Responsibility Across Individuals and Institutions, emphasizing public accountability and authority, and Collective Fear and Self-Protective Practices, reflecting affect-driven responses under uncertainty. Although negative emotions predominate, optimism appears conditionally, signaling coordination and recovery. Overall, disaster reporting constructs selfishness through integrated emotional–semantic patterns that position individual actions within broader social risk and collective responsibility.

## 1. Introduction

Disasters recurrently expose a fundamental tension between individual autonomy and collective risk management (Boin et al., 2017; Tierney, 2019). Although coordinated action is essential for mitigating shared threats, individuals often retain incentives to prioritize personal advantage, particularly under conditions of uncertainty and institutional ambiguity (Axelrod & Hamilton, 1981; Nowak, 2006). Classical social theory has long warned that intensified individualism can weaken civic obligation and erode concern for collective welfare (de Tocqueville, 1835/2003). These tensions become especially visible when policy responses depend on voluntary compliance, yet individual decision-making is shaped by perceived threat, scarcity, and responsibility (Feiock, 2013; Tierney, 2019). Within such contexts, media discourse renders self-interested behavior publicly visible and subject to evaluation, frequently through emotionally salient narratives that frame personal actions as socially consequential or morally contested (van Bavel et al., 2020; Drury et al., 2019).

Media discourse does not merely transmit information in disaster context but structures emotional meaning in ways that shape interpretations of responsibility and appropriate action. Prior research indicates that discrete emotions such as fear, disgust, and anger function as organizing mechanisms in communication, guiding how risk is interpreted, how accountability is assigned, and how responses to perceived threats are framed (Lerner et al., 2015; Nabi, 2002). From this perspective, emotional cues embedded in media texts operate as structured signals that align individual-level affect with broader narratives of social risk and collective vulnerability, providing a theoretical basis for examining emotion–topic configurations in disaster communication (van Bavel et al., 2020; Drury et al., 2019).

### 1.1. Emotional Cues in Disaster Media Discourse

Disaster-related responses, including selfish and prosocial behaviors, are not produced solely through deliberative reasoning (Slovic et al., 2005; LeDoux, 2000). Instead, they emerge from rapid effective processing of emotionally and semantically salient information (Pessoa, 2008; Lieberman, 2007), much of which is conveyed through media messages. From a media policy design perspective, this implies that communication strategies function not only as information delivery systems but also as emotional and cognitive regulators. Text-based analysis of media discourse therefore provides a means of identifying the affective cues and framing devices that are likely to engage cognitive–emotional processes underlying behavioral judgment and action (Entman, 1993; Forgas, 1995; Nabi, 2002).

Rapid emotional appraisal often precedes and constrains conscious decision-making, shaping how situations are interpreted and evaluated (Forgas, 1995; LeDoux, 2000; Pessoa, 2008). Contrary to popular disaster myths, this architecture does not inherently produce panic or social breakdown; cooperative and prosocial responses are common when institutional signals are coherent and trust is maintained. Panic-driven behavior is more likely to emerge under conditions of pre-existing social conflict, low institutional trust, and inconsistent or polarized media messaging—contexts that heighten threat perception and legitimize self-interested action (Alkalai Tavori & Adini, 2024; Drury et al., 2009; Tierney et al., 2006; Siegrist & Zingg, 2014).

Emotional cues embedded in media messages function as primary inputs that guide subsequent cognitive processing during crisis situations. Rather than bypassing cognition, affective signals such as fear, anger, and moral outrage shape how information is attended to, categorized, and evaluated, influencing judgments about responsibility, risk, and appropriate action (Slovic et al., 2005; Pessoa, 2008). Emotionally salient stimuli modulate higher-order cognitive functions by directing attention and constraining interpretive frames, thereby influencing deliberative decision-making and behavioral choice (LeDoux, 2000; Lieberman, 2007).

Media-generated emotional cues that foreground self-interest can influence how individuals interpret disaster situations through self-oriented rather than collective cognitive frames (Slovic et al., 2005; van Bavel et al., 2020). Messages emphasizing threat, scarcity, or norm violation tend to heighten vigilance and risk sensitivity, increasing the cognitive salience of self-protection and individual responsibility (Ansolabehere et al., 1991; Entman, 1993; Gross & D’ambrosio, 2004). Conversely, emotional cues associated with reassurance, efficacy, or shared purpose can support cognitive evaluations that favor coordination, trust in institutions, and compliance with collective measures (van Bavel et al., 2020; Drury et al., 2019). From a media policy perspective, this suggests that emotional cues are not merely expressive elements but operate as regulatory mechanisms that shape cognitive interpretations of crises, with direct implications for the design of communication strategies aimed at collective risk management.

### 1.2. Emotions and Associated Responses

Media messages that emphasize selfish or self-interested behaviors in disaster context tend to narrow the emotional range presented to audiences, foregrounding threat-related and self-protective emotions such as fear and anger while downplaying trust and collective orientation. Such emotionally structured framing prioritizes threat-sensitive and affective processing, shaping risk perception and behavioral responses in crisis contexts (LeDoux, 2000; Slovic, 2004; Pfaff, 2014).

Such emotionally structured framing prioritizes threat-sensitive and affective processing, shaping risk perception and behavioral responses in crisis contexts.

#### 1.2.1. Fear and Anxiety

Fear and anxiety are associated with self-interested responses by directing attention toward threat detection and immediate risk evaluation, while limiting deliberative processing (LeDoux, 2000; Slovic, 2004; Lerner & Keltner, 2001). This shift narrows attentional focus toward negative outcomes and immediate survival priorities, rendering self-protective actions more urgent and normatively defensible (LeDoux, 2000; Loewenstein et al., 2001; Lerner et al., 2015). Media reporting that emphasizes uncertainty and escalating self-interested actions can further amplify these effects, as observed during events such as the Hurricane Irma evacuation, where intensified fear coincided with competitive behaviors around resource acquisition (Wong et al., 2018).

#### 1.2.2. Anger and Moral Outrage

Anger and moral outrage can amplify self-interested responses during disasters by framing self-prioritization as a justified response to perceived injustice or institutional failure. Appraisal-based models suggest that anger arises when events are interpreted as controllable, blameworthy, and norm-violating, promoting approach-oriented responses and reduced cooperative restraint (Nabi, 2002; Richard et al., 2022). Media narratives emphasizing government incompetence or abandonment, as observed following Hurricane Katrina, have been shown to intensify such appraisals, contributing to declining trust in official guidance and greater reliance on self-directed or informal resource-seeking behaviors (Henkel et al., 2006; Tierney et al., 2006).

#### 1.2.3. Disgust and Aversion

Disgust is especially salient in disaster media narratives involving contamination because it triggers affective responses linked to disease avoidance, promoting withdrawal and boundary formation. From a behavioral perspective, disgust directs attention toward contamination cues and reinforces avoidance tendencies, reducing openness to social contact (Curtis et al., 2011). Empirical studies of the 2014 Ebola epidemic indicate that disgust-laden media framings reinforced avoidance of response teams and stigmatization of affected families, thereby undermining coordination and trust (Fairhead, 2016). While adaptive at the individual level, disgust-based processing can scale through media circulation to produce selfish behaviors by weakening solidaristic norms in crisis contexts.

#### 1.2.4. Sadness and Helplessness

Sadness and helplessness shape self-interested behavior in disasters in two distinct ways (Hobfoll et al., 2007; Lazarus, 1991). When amplified by loss-focused media messages, these emotions can deplete cognitive and motivational resources, reducing prefrontal regulatory engagement and biasing individuals toward short-term, self-protective responses such as withdrawal or stockpiling (Arnsten, 2009; Mullainathan & Shafir, 2013; Ochsner & Gross, 2005; Tierney, 2019). At the same time, sadness can also heighten sensitivity to vulnerability and shared loss, leaving its behavioral effects contingent on whether media framing emphasizes individual deprivation or collective recovery (Forbes, 2017). When media coverage foregrounds self-interested responses, sadness is more likely to translate into withdrawal and self-protection rather than empathy-based cooperation.

#### 1.2.5. Guilt and Shame

Guilt and shame regulate self-interested behavior by heightening sensitivity to anticipated social evaluation and reputational consequences (Fehr & Gächter, 2002). Guilt is associated with medial prefrontal and anterior cingulate engagement and can motivate reparative or prosocial responses when individuals perceive responsibility for others’ welfare; disaster studies document forms of survivor guilt that heighten concern about whether one has done enough to help (Aakvaag et al., 2014). In contrast, shame—associated with self-focused threat processing and withdrawal—can become defensive under accusatory media framing, prompting disengagement from guidance, resistance to external judgment, and reinforcement of self-protective narratives (Scheff, 2003). Under media messages that foreground selfishness or moral failure, these dynamics may suppress cooperative regulation while indirectly sustaining self-prioritizing behavior.

#### 1.2.6. Envy and Relative Deprivation

Envy and relative deprivation indicate that selfish behavior in disasters is shaped not only by scarcity but also by perceived distributional inequity. Media coverage that highlights uneven access to aid can activate social comparison processes, heightening sensitivity to relative disadvantage and perceived loss, which in turn encourages compensatory self-prioritizing responses (Van de Ven et al., 2012). Post-tsunami analyses of the 2004 Indian Ocean disaster show that perceived inequities in aid visibility and timing fostered intergroup resentment, weakening cooperation and legitimizing individual resource protection as a response to perceived unfairness (Telford et al., 2006).

#### 1.2.7. Relief and Selective Empathy

Relief in disaster contexts does not primarily diminish responsiveness to others’ suffering but rather reallocates empathic concern toward a narrower circle of beneficiaries (Cikara et al., 2011). Once individuals perceive themselves as safe, relief-related affect dampens threat monitoring and redirects empathic motivation toward close others, rendering assistance to distant or abstract victims discretionary (Hein et al., 2010). Media narratives emphasizing stabilization or recovery can reinforce this shift by signaling that acute risk has passed, thereby legitimizing reduced attention to broader collective needs. (Thomas et al., 2018). Behavioral research suggests that relief reduces the intensity of immediate affective responses while reinforcing valuation processes that privilege familiar targets, producing patterns consistent with selective empathy rather than compassion loss (Hein et al., 2010; Thomas et al., 2018).

Because affective and cognitive processes are activated and reinforced through public communication, media discourse provides a critical site for examining how selfishness is framed and evaluated in crisis contexts. Focusing on emotional cues and associated cognitive elements, the analysis conceptualizes selfishness not as a fixed moral category but as a layered, context-dependent linguistic construct shaped by emotional dynamics and situational narratives. Within crisis media discourse, selfishness emerges through patterned associations among emotional cues and thematic structures related to uncertainty, risk, and collective threat.

This study advances disaster communication research by conceptualizing selfishness as a discursive construct formed through integrated emotional–semantic configurations rather than as a fixed behavioral or moral category. It hypothesizes that specific emotion–topic alignments shape how media discourse frames responsibility and acceptable action in disaster contexts. It further hypothesizes that emotions such as fear, disgust, anticipation, and anger function as structuring mechanisms that organize these framings. These mechanisms enable media discourse to linguistically connect representations of individual-level affect with broader narratives of social risk and collective vulnerability.

Building on this framework, the study aims to linguistically explore and visually demonstrate how selfishness is emotionally constructed in disaster-related media discourse by identifying dominant emotional patterns, extracting thematic structures through topic modeling, and examining their interaction within an integrated emotion–topic framework. It further aims to explain how these emotional–semantic configurations, as the underlying structure of selfishness-focused media messages, shape interpretations of responsibility and response within broader narratives of collective disaster.

## 2. Methods

The analysis followed a four-step workflow. First, news articles were divided into individual sentences and short overlapping segments to capture both detailed expressions and their immediate context. Second, each unit was assessed to estimate the presence of a range of emotions. Third, the texts were analyzed to identify the main themes running through the corpus. Fourth, the emotional and thematic results were combined to examine how different emotions align with specific themes. This approach provides an overview of how selfishness is represented in disaster-related media discourse from both emotional and thematic perspectives.

### 2.1. Data Selection

This selection strategy reflects methodological research showing that the adequacy of automated text analysis depends not solely on corpus size, but on alignment with the research question—particularly the diversity of behavioral forms, discursive contexts, and social evaluations represented, as well as the granularity of analytical units employed (Grimmer & Stewart, 2013; Lacy et al., 2015). The dataset was constructed through a criterion-based, purposive sampling process conducted over a two-year period to capture information-rich cases in which individual-level selfish or norm-violating behavior is explicitly framed in disaster contexts. The dataset was compiled from credible news articles that frame self-interested, responsibility-avoiding, or norm-violating behaviors in disaster contexts as sources of social conflict and public risk. Articles were systematically collected by tracing coverage backward from 2024 across internationally recognized news organizations characterized by professional editorial standards and broad public reach.

During this process, substantial redundancy was observed across articles, particularly among reports covering the same event or reproducing similar narrative and linguistic structures. To preserve discursive variation and avoid overrepresentation of repeated patterns, the sampling approach limited inclusion to a small number of representative articles per event and excluded closely overlapping texts, as well as articles primarily focused on institutional misconduct rather than individual behavior.

Cross-disaster and cross-period pooling is analytically necessary for this study, as media discourse is organized through recurrent framing logics and discursive formations that are reproduced and stabilized across events and over time, enabling the identification of higher-order patterns and reducing the influence of event-specific contingencies that would not be observable within single-event or time-bound analyses (Entman, 1993; Fairclough, 1995). This approach is also warranted by the relative scarcity of articles that explicitly focus on selfish or self-interested behavior, making cross-contextual aggregation necessary to capture sufficient instances of such discursive constructions. An initial pool of 57 articles was identified; editorials, duplicated reports, articles primarily focused on photographic content, and texts lacking sufficient narrative depth for systematic analysis were excluded. The final dataset consisted of 12 text-centered articles suitable for text analysis, as summarized in Table 1.

The dataset was constructed through a criterion-based, purposive sampling process designed to assemble information-rich cases in which selfish or norm-violating behavior is explicitly framed in disaster-related news coverage (Grimmer & Stewart, 2013; Lacy et al., 2015). This sampling approach supports the conceptual focus of the study; however, it does not in itself establish the statistical stability of topic model estimates. Accordingly, the LDA results are interpreted as exploratory summaries of recurrent lexical patterns within this corpus rather than as stable or generalizable topic structures, and the two-topic solution is treated as a provisional interpretive device within the present dataset (Blei et al., 2003; Grimmer & Stewart, 2013). Given the constrained corpus size, the stability of these topic structures cannot be fully established (Tang et al., 2014; Sbalchiero & Eder, 2020). Inter-rater reliability for article selection was high (Krippendorff’s α = 0.91). Disagreements occurred in three cases and were resolved through discussion until consensus was reached.

### 2.2. Data Processing

The *unit of analysis* was defined to balance contextual continuity with analytical granularity for emotion and topic analysis. Unit definition and preprocessing were treated as core data-processing decisions, given their influence on interpretive outcomes beyond document-level aggregation (Van Atteveldt et al., 2021). Emotional and evaluative expressions, particularly those related to self-interested behavior, often unfold across contiguous discourse rather than isolated sentences, making unit construction a critical methodological consideration (Grimmer & Stewart, 2013; Hase, 2023).

The *corpus* was constructed by compiling approximately 4,000 words across over 220 sentences from selected media articles. Headlines and body texts were extracted and organized into an article-level data frame containing the article title, full text, and a unique article identifier. This structure ensures traceability between analytical units and their source texts throughout the analysis.

*Text segmentation* was performed at the sentence level, followed by the construction of overlapping multi-sentence units. Article texts were segmented into sentences and expanded into a sentence-level data frame, with article identifiers retained for contextual reference. A fixed-length sliding-window approach grouped sequences of three consecutive sentences into a single analytical unit, preserving local semantic context while generating multiple observations (Jurafsky & Martin, 2009). This approach enhances the identification of emotional and thematic patterns without relying on full document aggregation (Grimmer & Stewart, 2013; Stevens et al., 2012).

### 2.3. Sentiment Analysis

When individuals are exposed to disaster-related information, emotional responses are rarely discrete but instead involve overlapping affective states—such as fear, anger, sadness, trust, and optimism—that jointly shape risk per3ception and behavioral tendencies (Buechel & Hahn, 2018). This analytic work employs an eleven-category emotion framework to capture these co-occurring patterns, which is well suited to disaster-related media discourse characterized by mixed and layered emotional cues (Mohammad, 2020).

*Emotion classification* was performed using the TweetNLP implementation of a transformer-based model based on the RoBERTa architecture. The model estimates probability distributions across eleven discrete emotion categories—anger, anticipation, disgust, fear, joy, love, optimism, pessimism, sadness, surprise, and trust—reflecting widely used multidimensional approaches in computational emotion research (Camacho-Collados et al., 2022; Liu et al., 2019; Mohammad, 2020). However, because the model was trained on social media data, its application to formal news discourse required empirical validation. A random sample of 50 analytical units from the news corpus was independently annotated by two human coders with expertise in text analysis using the same 11-category emotion framework employed in the computational analysis. Intercoder reliability was assessed using *Krippendorff’s alpha* for nominal data (Krippendorff, 2018), yielding *α* = 0.69, indicating a moderate to substantial level of agreement. Disagreements were resolved through discussion to produce a final human-coded label for each unit.

Model–human agreement was then evaluated by comparing the model’s highest-probability emotion label with the final human-coded label using overall accuracy and Cohen’s kappa (Cohen, 1960; Artstein & Poesio, 2008). The results showed an overall accuracy of 0.72 and a *Cohen’s kappa of* 0.68, indicating moderate to substantial agreement beyond chance. While these values reflect a meaningful level of alignment, the magnitude of agreement remains moderate, which is consistent with the relatively small size of the validation sample. These findings indicate that the model reflects general emotional patterns in the text while maintaining an appropriate level of caution in interpretation.

*Emotion estimation* was conducted at the sentence level to quantify overall emotional distributions in the corpus. For each unit, the model outputs a probability vector across all emotion categories, with the highest-probability category assigned as the primary label while retaining full probability distributions for analysis. This probabilistic representation captures overlapping emotional signals common in media texts (Buechel & Hahn, 2018). Accordingly, emotion outputs are interpreted as probabilistic indicators contributing to pattern detection, rather than as definitive labels assigned to individual sentences (Grimmer & Stewart, 2013).

To enhance robustness, *emotion estimation* was extended to multi-sentence units using a sliding-window approach. Groups of two to three consecutive sentences were treated as a single unit to reduce sentence-level variability and contextual fragmentation (Buechel & Hahn, 2018; Grimmer & Stewart, 2013). Emotion distributions were compared across sentence-level and window-level units to identify consistent patterns across analytical scales (Roberts et al., 2014). This multi-level aggregation reduces the influence of potential misclassification at the sentence level and allows more stable emotional patterns to emerge across the corpus. However, it is not treated here as a substitute for domain validation; rather, the added human-coding step provides a separate empirical check that is directly relevant to the news-text context.

*Emotion–topic relationships* were examined by integrating emotion classification with LDA (Latent Dirichlet Allocation) topic modeling. Each multi-sentence unit was assigned a representative emotion based on the highest predicted probability, and corresponding topic–word weights were extracted using LDA (Blei et al., 2003; Grimmer & Stewart, 2013). These weights were aggregated by emotion category to identify emotion-linked semantic patterns.

*Visualization* was employed to represent emotion-specific semantic patterns. For each emotion, salient terms were identified based on aggregated topic–word weights and presented using bar charts and emotion–term network graphs. Edge thickness in the network visualizations indicates relative importance, supporting clearer interpretation of emotion–term relationships.

### 2.4. Topic Modeling

Model Selection and Topic Labeling were conducted using LDA on the full corpus (Blei et al., 2003), following standard preprocessing procedures. Models with varying numbers of topics were evaluated using coherence scores, and a two-topic solution was selected based on coherence, interpretability, and corpus size (Röder et al., 2015). Given the small, purposively selected corpus, coherence scores were treated as heuristic indicators rather than as evidence of topic stability. Accordingly, the resulting two-topic solution is interpreted as preliminary and exploratory (Roberts et al., 2014).

Topic keyword extraction and visualization were conducted to support interpretation. For each topic, the ten highest-weighted keywords were identified and visualized using bar charts and network graphs to represent relative importance and topic–word associations (Stevens et al., 2012).

### 2.5. Integration: Topic–Emotion Mapping

Topic–emotion mapping was conducted to examine the alignment between thematic structures and emotional patterns. Each three-sentence unit was associated with (1) a topic probability distribution generated by the LDA model and (2) an emotion probability distribution produced by the emotion classifier.

Rather than assigning each unit to a single topic, a probabilistic (soft) assignment approach was adopted, allowing each unit to contribute to multiple topics in proportion to its topic probabilities. This approach accounts for the presence of mixed thematic content within textual units. Topic-level emotion profiles were computed using weighted aggregation. For each topic k and emotion e, the aggregated value was calculated as:

*Emotion*(*k*, *e*) = *Σ_d* [*θ*(*d*, *k*) × *p*(*d*, *e*)]/*Σ_d* [*θ*(*d*, *k*)]

where *θ*(*d*, *k*) denotes the probability of topic k in unit d, and *p*(*d*, *e*) denotes the probability of emotion e in the same unit. This formulation ensures that contributions from each unit are proportionally weighted and that the resulting emotion profiles represent averaged patterns across the corpus.

**Figure 1:**
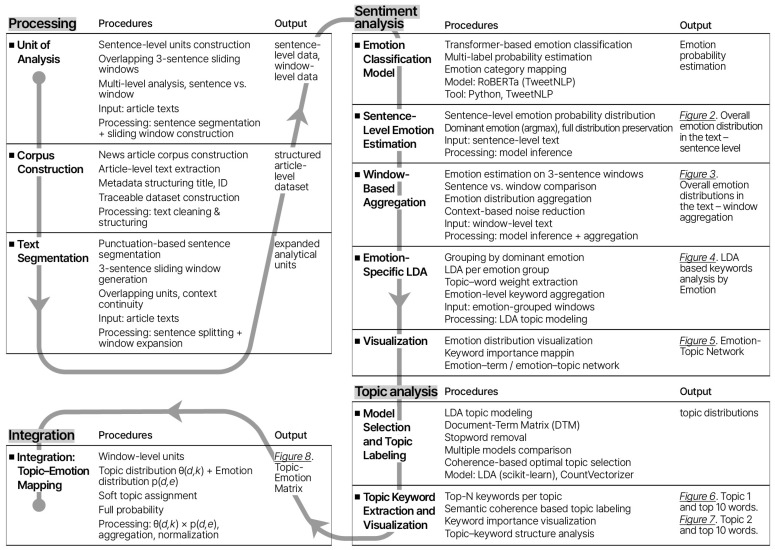
Overview of the Text Analysis Procedure.

No thresholding or filtering was applied to emotion probabilities prior to aggregation. The full probability distributions were retained, allowing each unit to contribute across multiple emotional dimensions. The resulting topic–emotion matrix enables comparison of emotional distributions across topics. An overview of the analytical workflow is provided in Figure 1.

## 3. Results

### 3.1. Sentiment Analysis

**Figure 2:**
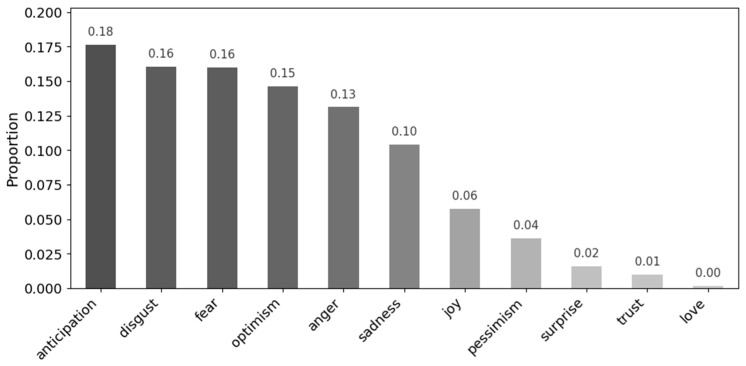
Overall emotion distribution in the text—sentence level.

The results first describe the overall distribution of emotions associated with media messages framing selfish behavior. Emotions were classified at the sentence level, and the proportion of each emotion represents its relative prevalence across the corpus (see Figure 2). The validity of the classification in the news-text context was assessed through a separate human-annotation procedure (see Section 2.3), which indicated a moderate to substantial level of agreement between model predictions and human judgments.

Anticipation emerges as the most prevalent emotion, followed by disgust and fear. This pattern is observed at the sentence level and reflects the forward-looking orientation of the media texts, which emphasize the projected consequences of selfish behavior, including increased infection risk, policy disruption, and broader social instability. Such coverage frequently relies on predictive or cautionary framing—highlighting potential escalation and future harm—thereby situating selfish actions within narratives of anticipated risk rather than immediate outcomes.

Disgust and fear, the next most prevalent emotions, are closely associated with moralized risk appraisal. These emotions are primarily elicited by media portrayals of behaviors such as panic buying and other forms of individualistic conduct framed as endangering others. In this context, disgust reflects moral aversion to perceived norm violations, whereas fear signals perceived threats to collective safety.

Optimism appears alongside fear and anger as a prominent emotional category, occupying an intermediate position between threat-oriented and blame-oriented responses. This pattern suggests that media discourse does not move directly from fear to anger, but incorporates resolution-oriented affect. Anger remains a high-profile emotion, reflecting moral evaluation and responsibility attribution, while optimism signals expectations of correction or containment within the same discursive space. Anger remains a high-impact emotion in the discourse, but it is typically articulated through risk-oriented and norm-enforcing framings rather than explicit moral outrage.

**Figure 3:**
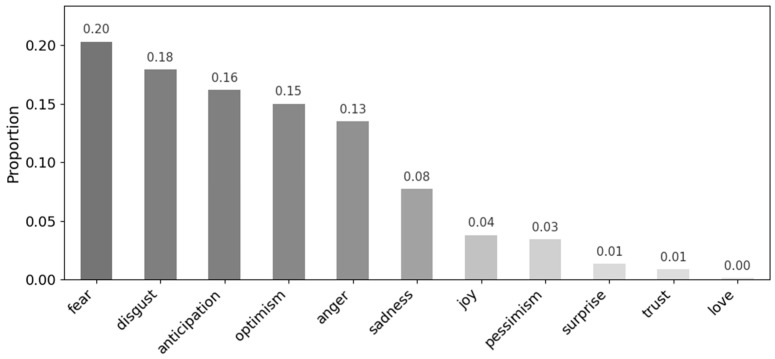
Overall emotion distributions in the text—window aggregation.

To enhance robustness and mitigate sentence-level dependence, three adjacent sentences were aggregated into a single analytic unit and analyzed using the same modeling approach; the resulting distributions are presented in Figure 3.

Under this multi-sentence configuration, the emotional distribution shifted relative to the sentence-level analysis, with fear (20.3%), disgust (18.0%), and anticipation (16.0%) emerging as the most dominant emotions. This shift indicates that emotions are interpreted more contextually at the multi-sentence level, such that positive expressions within a single sentence may be attenuated or overridden by negative cues in surrounding text.

Because the corpus contains frequent references to disasters, infectious diseases, and broader societal risks, the multi-sentence analysis tends to integrate these cues into a more unified threat context, which the model predominantly classifies as fear. By contrast, at the sentence level, forward-looking or cautionary statements were more often categorized as anticipation. When three adjacent sentences are analyzed jointly, however, extended sequences of risk description, social criticism, or institutional failure outweigh isolated predictive framing, shifting emotional classification from anticipation toward fear.

### 3.2. Emotion-Specific LDA Topic Modeling

**Figure 4:**
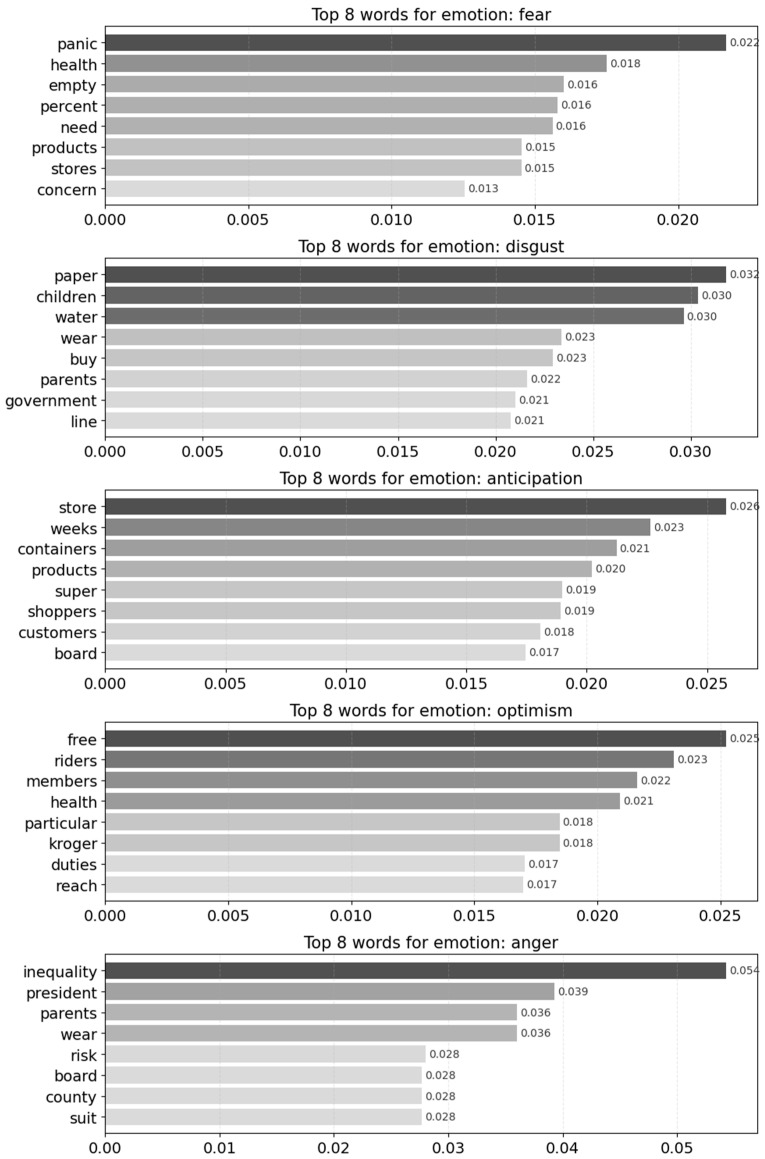
LDA based keywords analysis by Emotion.

**Figure 5:**
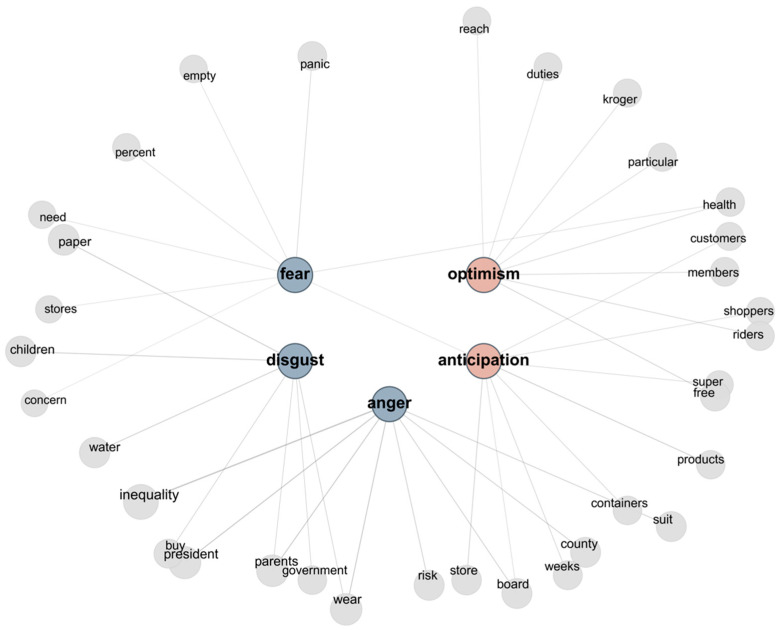
Emotion–Topic Network of Selfishness Framing.

Results from the emotion-specific LDA topic modeling indicate distinct semantic patterns associated with dominant emotional categories in media messages framing selfish behavior. Topics were examined in relation to emotion-linked keyword distributions, allowing for identification of how specific emotional states align with recurring semantic themes. Bar charts (see Figure 4) illustrate the relative prominence of key terms within each emotion, while network graphs (see Figure 5) depict the relational structure among keywords, highlighting the discursive patterns through which selfish and self-interested responses are constructed in disaster-related media discourse.

Fear is associated with terms such as panic and health, indicating heightened concern over public hygiene and collective risk, as well as words like *empty*, *need*, *products*, and *stores*, which reflect anxieties about systemic instability and widespread scarcity rather than isolated incidents. The presence of quantified terms, such as *percent*, further suggests that fear functions not only as an affective response but also as a collective risk frame shaped by perceived vulnerabilities in supply and distribution systems.

Disgust is associated with consumption-related terms such as *paper*, *water*, and *buy*, alongside references to *children*, *parents*, and *government*. This pattern links essential-goods purchasing to moral evaluation, particularly when caregiving roles are invoked. The inclusion of governmental references suggests that such behaviors are framed not only as individual failures but also as consequences of regulatory or coordination breakdowns, positioning disgust as a mode of moral condemnation toward socially irresponsible conduct.

Anticipation, a forward-looking affect, is associated with stockpiling and supply-related terms such as *store*, *containers*, *shoppers*, *products*, and *weeks*. This lexical pattern reflects expectations of future scarcity and systemic disruption. In this context, anticipation indexes uncertainty and collective anxiety surrounding the potential escalation of hoarding behavior and supply-chain instability, rather than positive or hopeful outlooks.

Optimism co-occurs with terms related to *membership*, *duty*, *health*, and *reach*, alongside references to institutional actors such as major retailers. Rather than signaling unqualified positivity, this configuration suggests conditional confidence in collective responsibility and institutional capacity to manage disruption, even as awareness of opportunistic or free-riding behavior remains salient.

Anger is most strongly associated with terms such as *inequality*, *president*, *board*, *county*, and *suit*, indicating that it is oriented primarily toward policy decisions and institutional authority rather than interpersonal misconduct. The co-occurrence of household-related terms, including *parents*, *wear*, and *risk*, points to tensions at the intersection of private decision-making and collective safety, while references to inequality foreground perceptions of uneven policy impacts. The emotion of anger reflects concerns about political accountability, institutional fairness, and unequal risk distribution, under conditions that may intensify individual selfishness.

### 3.3. Topic Analysis

**Figure 6:**
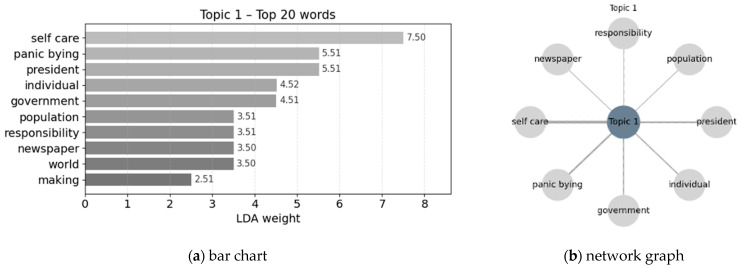
Topic 1 and top 10 words.

LDA topic modeling was applied to identify provisional thematic structures within the corpus, with the ten highest-weighted terms extracted for each topic. These topic labels are interpreted as summaries of patterns observed in the present dataset rather than as stable latent structures of broader disaster-media discourse. Results are presented using bar charts to indicate relative term importance and network graphs to visualize structural relationships among key terms, with edge thickness reflecting relative weight. The network visualizations complement the bar charts by emphasizing the internal word structure of each topic through reduced term sets and topic-specific layouts (see Figure 6).

Topic 1 centers on the tension between individual responsibility and institutional accountability. Keywords such as *individual*, *responsibility*, and *self-care* emphasize personal choice while situating it within a framework of collective consequence. References to *government* and *president* anchor these behaviors in broader debates over political leadership and institutional response, while terms such as *population* and *panic buying* extend the issue to the level of societal risk. The presence of *newspaper* underscores the mediating role of news coverage in circulating and stabilizing these interpretations. Overall, this topic frames selfish behavior not as private misconduct but as a publicly contested issue shaped by responsibility attribution, institutional authority, and social evaluation.

**Figure 7:**
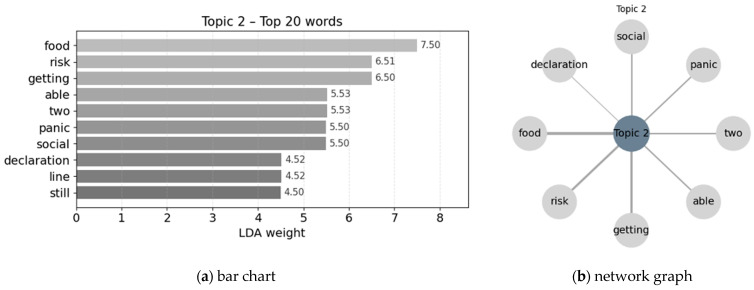
Topic 2 and top 10 words.

Topic 2 focuses on everyday self-protective behaviors shaped by shared fear under crisis conditions (see Figure 7). Keywords such as *panic*, *social*, *food*, *getting*, *line*, and *still* capture collective anxiety and routine practices—including waiting, congestion, and stockpiling—through which individuals seek to secure essential resources amid uncertainty. References to *risk*, *able*, and *two* reflect ongoing assessments of vulnerability and constrained capacity, indicating that these behaviors are oriented toward managing perceived risk rather than expressing stable dispositional tendencies. The inclusion of *declaration* situates individual actions within broader social conditions and official communications that activate or legitimize such responses. Overall, this topic characterizes these behaviors as context-dependent responses to instability and perceived threat.

Topics 1 and 2 delineate a clear distinction between normative–institutional and situational–affective framings of self-interested behavior in disaster-related media discourse. The first topic reflects *Responsibility Across Individuals and Institutions*, foregrounding politically and normatively framed evaluations of individual behavior. It emphasizes debates over responsibility, rights, and institutional authority, and is more closely associated with cognitively oriented reasoning about norms, accountability, and governance. Individual actions are interpreted in relation to broader political leadership and institutional response, situating behavioral evaluation within public discourse on collective responsibility. The second topic reflects *Collective Fear and Self-Protective Practices*, capturing situationally driven behaviors shaped by uncertainty, fear, and perceived resource scarcity. This topic highlights routine self-protective actions that emerge under crisis conditions, such as securing essential goods and managing perceived risk, and is strongly aligned with affectively driven responses to instability.

### 3.4. Topic–Emotion Mapping Based on LDA

**Figure 8:**
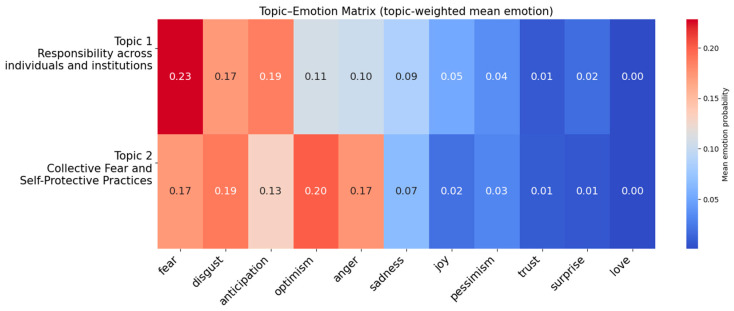
Topic-Emotion Matrix.

Each row represents one of the two thematic topics extracted through LDA, Topics 1 and 2, and each column corresponds to one of the eleven emotions ranging from optimism to love predicted by the model. The value in each cell indicates the weighted average emotional score, calculated by weighting emotion probabilities by the topic probability of the paragraphs assigned to each topic. Based on the results of the LDA topic modeling, Figure 8 illustrates how each topic is differentially associated with specific emotional profiles.

The heatmap shows that fear and disgust account for a substantial proportion of the emotional weight in both topics, although their overall emotional configurations differ. Topic 1, *Responsibility Across Individuals and Institutions*, is characterized by relatively elevated levels of fear, anticipation, disgust, and sadness. This emotional profile suggests that media discussions centered on responsibility and accountability are accompanied by multiple, overlapping negative affective responses. The co-occurrence of fear and anticipation reflects uncertainty surrounding institutional decision-making and future outcomes, while the presence of disgust and sadness points to broader affective reactions such as frustration, moral disapproval, or emotional fatigue. Evaluations of individual behavior within this topic are embedded in a discourse marked by sustained negative emotion and ongoing contestation over institutional authority and social responsibility.

Topic 2, *Collective Fear and Self-Protective Practices*, likewise displays fear as the salient emotion, but with a distinct emotional pattern. In this topic, fear, disgust, and anger are more evenly distributed and are accompanied by a noticeable level of optimism. This configuration indicates that emotional responses are not confined to negative affect alone but include affective states associated with adaptation and action. In this context, fear appears to be closely associated with behavioral responses, which may facilitate a partial restoration of perceived control, as evidenced by the concurrent presence of optimism in the emotional profile.

Based on the topic–emotion matrix integrating emotion classification with LDA-derived topics, *Responsibility Across Individuals and Institutions* exhibits a pattern of accumulated and persistent negative emotion associated with extended public debate and responsibility attribution. By contrast, *Collective Fear and Self-Protective Practices* reflects a shift from negative affect toward short-term emotional regulation through immediate action. While emotional responses in the first topic remain embedded in institutional and normative conflict, those in the second topic suggest an action-oriented emotional sequence that begins with fear and moves toward provisional control or reassurance.

## 4. Discussion

The findings of this study do not demonstrate direct effects on individual behavior, but instead explore how behavior is constructed and interpreted within media discourse. This distinction is critical for situating the analysis within a discursive framework. In disaster contexts, discursive evaluations of responses are reconfigured, situating self-interested actions within broader expectations of social responsibility and shaping these assessments through underlying emotional processes.

As hypothesized, emotion–topic alignments structure interpretations of selfish behavior, with fear, disgust, anticipation, and anger functioning as organizing mechanisms that inform judgments of responsibility and acceptable action. Consistent with the study’s objectives, selfishness emerges in disaster-related media discourse through the convergence of emotional and thematic patterns, where emotional–semantic configurations shape how responsibility and response are interpreted within evolving narratives of collective disaster.

Selfishness is consistently constructed within predominantly negative affective contexts, with its emotional configuration varying across analytical scope. At the sentence level, anticipation emerges as the most prevalent emotion, followed by disgust and fear. This pattern indicates that media discourse frames selfish behavior in forward-looking terms, emphasizing projected consequences such as escalating health risks, policy breakdowns, and collective vulnerability. Such future-oriented framing positions selfishness not simply as immediate misconduct but as a catalyst for potential systemic harm.

The co-occurrence of disgust and fear suggests that selfish behavior is evaluated through a dual affective structure combining moral judgment and threat perception. Disgust functions as a mechanism of moral boundary construction, marking certain behaviors as socially unacceptable, while fear reflects anxiety about contagion, scarcity, and loss of collective control. The relatively limited presence of anger indicates that selfishness is less frequently framed as an object of direct moral outrage and more as a source of diffuse social risk. Notably, optimism exceeds anger in prevalence, indicating that media narratives incorporate elements of correction, recovery, and collective adjustment alongside moral evaluation.

When the analytical window expands to multi-sentence units, fear becomes the dominant emotion, surpassing anticipation. This shift highlights the importance of narrative context in shaping emotional interpretation. While isolated sentences emphasize anticipated risk, extended passages situate selfish behavior within broader crisis narratives characterized by escalation, uncertainty, and systemic instability. In this context, selfishness is embedded within sustained fear-based storylines concerning societal breakdown, resource insecurity, and institutional strain. These patterns suggest that selfishness is not associated with discrete emotional reactions but is embedded within enduring affective structures that amplify collective anxiety and perceived threat.

Emotion-specific keyword patterns further indicate that selfishness is articulated across multiple emotional domains, each shaping distinct interpretive frames. Rather than merely accompanying representations of behavior, emotions function as structuring mechanisms that organize how selfishness is rendered meaningful within crisis discourse.

The emotional patterns identified in this analysis suggest that selfishness is not evaluated through a single moral lens but is differentially structured across distinct affective domains that organize its meaning in crisis contexts. Fear-oriented patterns position selfishness as a risk multiplier, where individual actions are interpreted through their capacity to destabilize an already fragile collective system, shifting evaluation away from moral transgression toward the amplification of shared vulnerability and uncertainty. Disgust, by contrast, stabilizes moral meaning by delineating boundaries between legitimate and illegitimate forms of consumption, operating as a normative sorting mechanism that renders selfish behavior visible as a violation of social obligations, particularly in relation to caregiving and access to essential goods.

Anticipation introduces a forward-looking interpretive frame in which selfish actions are situated within calculations shaped by uncertainty, recasting behaviors such as stockpiling as attempts to regain control over an unstable future rather than as purely ethical failure. Optimism, while limited and conditional, functions to contain these disruptions by reframing selfishness as a temporary deviation within a system expected to recover through institutional coordination, thereby sustaining expectations of regulation and cooperation. In contrast, anger reorients evaluation from individual behavior to institutional conditions, politicizing selfishness by embedding it within narratives of governance, inequality, and policy inconsistency.

These patterns indicate that emotions operate not merely as reactions but as structuring mechanisms that differentially frame selfishness as risk, norm violation, adaptive response, temporary disruption, or systemic failure, depending on the affective configuration through which it is articulated.

Emotion patterns in this study highlight how selfishness in disaster-related media discourse is not framed through a single perspective but through two distinct interpretive logics. *Responsibility Across Individuals and Institutions* presents selfishness as a matter of public judgment, where individual actions become a basis for evaluating authority, fairness, and institutional legitimacy. In this framing, selfish behavior is less about the act itself and more about what it reveals regarding governance and collective obligation, allowing it to serve as a focal point for broader concerns about leadership, accountability, and the distribution of risk.

By contrast, *Collective Fear and Self-Protective Practices* frame selfishness as a situational response to uncertainty. Here, self-protective actions are understood as practical responses to perceived threat, shaped by conditions of scarcity and urgency rather than by moral intent. These behaviors are not necessarily treated as violations of collective norms but are instead normalized as ways of coping with instability, especially when public messaging amplifies a sense of risk and immediacy.

This contrast reveals that media discourse moves between judging selfishness and explaining it, shifting how responsibility and response are interpreted in crisis contexts. Framing selfishness solely as a moral failure overlooks the conditions under which such behavior emerges, while situational interpretations bring attention to how uncertainty, risk perception, and institutional signals influence public responses. The distinction therefore highlights the limits of purely moral evaluations and underscores the importance of considering how context shapes the meaning of individual action.

Emotional patterns reinforce this divide. *Responsibility Across Individuals and Institutions* is associated with a sustained negative tone, where concern, disapproval, and uncertainty accumulate, reflecting ongoing tension around accountability and unresolved outcomes. In contrast, *Collective Fear and Self-Protective Practices* shows a more varied emotional profile in which concern is accompanied by moments of adjustment and partial stabilization. This pattern suggests that while fear may initially drive self-protective behavior, subsequent actions can ease uncertainty to some extent, allowing for a limited sense of regained control even within continuing disruption.

Effective crisis communication therefore depends not only on informational accuracy but also on the emotional structures through which responsibility and legitimacy are articulated in media texts. Communication approaches that mitigate uncertainty, foreground institutional capacity, and emphasize shared responsibility may be more conducive to sustained compliance and cooperation than those relying on moralized emotional intensification.

Interpretations of selfish behavior are shaped by underlying emotional conditions and structural contexts that jointly inform perception and response. Crisis communication and policy interventions are therefore likely to be more effective when they are attentive to the emotional dynamics of disaster contexts and seek to mitigate uncertainty and perceived risk while reinforcing shared norms of responsibility, rather than relying primarily on moral exhortation. Emotion should be understood not as a variable subject to direct manipulation, but as a constitutive element of crisis dynamics. Emerging at the individual level and circulating through media and public discourse, emotional responses structure collective interpretation and behavioral coordination. Recognizing emotion as an integral component of crisis communication is thus essential for understanding and managing cooperative responses during disasters.

## 5. Conclusions

Disaster-related media do not merely report self-interested behavior but construct it through patterned emotional–semantic configurations that shape how such actions are understood and evaluated. Across contexts, fear and disgust emerge as dominant signals, while anticipation organizes projections of potential harm and conditional optimism appears within narratives of coordination and recovery.

The amplification of disgust in media discourse emerges as a particularly salient pattern, suggesting a possible communicative function of curbing the spread of such behaviors by leveraging emotional responses. At the same time, this interpretation remains tentative and requires further empirical investigation. In addition, the findings indicate that emotional responses to selfish behavior are not uniformly aligned with specific message frames but instead constitute a more complex and differentiated affective structure than initially anticipated.

Two distinct yet interrelated discursive configurations become evident: *Responsibility Across Individuals and Institutions* and *Collective Fear and Self-Protective Practices*. The former situates selfishness within ongoing negotiations of responsibility, authority, and accountability, whereas the latter reflects situational responses to uncertainty in which fear-oriented reactions are accompanied by short-term efforts to restore control. Together, these configurations indicate that selfishness is not treated as a stable moral category but as a context-dependent construct shaped by emotional structuring and narrative positioning.

Attention to these patterns highlights how media discourse organizes interpretations of responsibility and action through emotional cues embedded in broader narratives of collective vulnerability. The focused and purposively selected nature of the dataset supports the conceptual scope of the study, while not establishing the stability of a probabilistic topic model at this scale (Tang et al., 2014; Sbalchiero & Eder, 2020). Accordingly, the findings should be interpreted as exploratory within-corpus patterns rather than as stable or generalizable structures, or as classifications applicable beyond the present dataset, identifying cross-contextual discursive regularities rather than context-specific effects, and reflecting aggregated emotional–discursive patterns rather than precise sentence-level classifications. Extending this approach to more varied datasets and comparative contexts, including non-selfish behavioral framings, would further clarify how these emotional–semantic structures operate under different communicative conditions and how they may vary across disaster types, temporal settings, and institutional contexts; human-coding validation and model–human agreement assessment were conducted on a sample of 50 analytical units to provide an empirical check on the emotion classification. While this process strengthens the methodological grounding of the study, further validation using larger samples and additional comparative designs would support a more comprehensive assessment of the robustness of this approach.

While the controversial nature of selfishness as a dimension of human behavior extends beyond the scope of this study, the patterns identified here suggest a structured way of understanding how such behavior is represented and interpreted in crisis contexts, offering a basis for future research to examine how these underlying configurations shape responses across different social and communicative settings.

## Figures and Tables

**Table 1:** Overview of selected media articles.

ID	Year	Title	Source
01	2012	After Sandy, officials are wondering what it takes to get people to heed evacuation orders	Global News
02	2013	Biggest fines for gouging after Sandy imposed on New York fuel sellers	Associated Press/Reuters
03	2015	Nepal earthquake: Government to investigate profiteering	The Guardian
04	2017	3 Texas businesses sued for alleged price gouging during Harvey.	WCNC
05	2018	Hurricane Michael leaves ‘unimaginable destruction’	BBC News
06	2018	Homeless “hero” jailed for stealing from British bomb victims	Reuters
07	2020	America’s selfish individualism has become a death cult in the COVID-19 era	Business Standard News
08	2020	Florida parents sue school board over mandate that requires students to wear masks	NBC Philadelphia
09	2021	McConnell strives to counter bad advice to boost U.S. Republican vaccination rate	Reuters
10	2022	“Lowest form of scum”: Lee County deputies arrest Orlando men for allegedly looting after Hurricane Ian	FOX 13 News
11	2024	Rush on gas & groceries causes some shortages, long lines ahead of Hurricane Milton	News4JAX
12	2024	Thousands flee Hurricane Milton, causing traffic jams and fuel shortages	Reuters

## Data Availability

Data are available by request from the corresponding author subject to embargo.
